# Supplementary material for: Integrative Analysis of Multi-Omics Data Based on Blockwise Sparse Principal Components
Source: Int J Mol Sci. 2020 Nov 2;21(21):8202. doi: 10.3390/ijms21218202 (PMC7663540; doi:10.3390/ijms21218202)
Supplement: Supplementary file 1 [file ijms-21-08202-s001.pdf]

Supplementary Table S1. Loadings of the first sPCs, by sPCA, for selected blocks.

	DNA						mRNA						miRNA	
D4	ALK_E183_R	5.99E-01	D6	BCL3_E71_F	7.86E-01	MR4	GLP1R	6.95E-01	MR5	PTPRZ1	6.38E-01	MI3	hsa-miR-602	1.00E+00
	KRAS_P651_F	5.27E-01		COL6A1_P425_F	4.51E-01		NCR2	6.33E-01		EPB41L4B	-4.80E-01		kshv-miR-K12-9	2.41E-05
	IFNGR2_E164_F	3.95E-01		MCAM_P265_R	3.22E-01		KIAA0953	2.38E-01		JAM3	4.43E-01		hsa-miR-563	6.64E-06
	RASA1_E107_F	3.26E-01		INHA_P1144_R	1.98E-01		SLC9A3	1.55E-01		GRIA3	2.81E-01	MI5	hsa-miR-648	7.30E-01
	CCKBR_P361_R	2.89E-01		CD34_E20_R	1.79E-01		CNGB1	1.51E-01		MAPK13	-2.70E-01		ebv-miR-BART8-3p	5.18E-01
	FGF9_P862_R	8.41E-02		EPS8_E231_F	4.97E-02		AVPR1A	8.37E-02		RBM35B	-9.08E-02		hsa-miR-604	4.32E-01
	PTCH_E42_F	7.63E-02		IGSF4C_E65_F	1.67E-02		MYLC2PL	5.68E-02		SOX2	6.79E-02		kshv-miR-K12-2	1.11E-01
	APC_E117_R	6.75E-02		BCAM_P205_F	1.62E-02		NMUR1	4.41E-02		SPINT1	-4.52E-02		hsa-miR-658	2.39E-04
	WNT5A_E43_F	3.28E-03		KLF5_E190_R	1.30E-03		BTNL8	3.05E-02		CLIP3	4.08E-03		hsa-miR-629	9.35E-05
	EFNB3_E17_R	6.55E-04		LAMB1_E144_R	7.45E-04		FUT6	9.44E-03		CLDN3	-2.53E-03		hsa-miR-639	8.90E-05
	CDKN1C_P6_R	3.00E-04		ITGB1_P451_F	7.35E-04		PRO1768	3.63E-03		BHLHB9	1.35E-03		ebv-miR-BART17-5p	7.05E-05
	GAS1_P754_R	5.73E-05		PCGF4_P760_R	6.28E-04		GNRHR	1.76E-03		ABHD4	1.22E-03	MI7	hsa-miR-17-5p	1.00E+00
	CDKN2B_E220_F	3.37E-05		FGFR1_E317_F	3.75E-04		ADRB3	1.11E-03		BAI3	7.42E-04		hsa-miR-106a	1.17E-02
D5	FES_E34_R	7.51E-01		DAB2IP_E18_R	1.45E-04		PCTK2	-7.97E-04		LLGL2	-4.38E-05		hsa-miR-20a	6.07E-03
	FAS_P65_F	4.00E-01		KLF5_P13_F	1.27E-05		PDCL3	-7.89E-04		PSRC1	2.82E-05		hsa-miR-20b	6.02E-04
	GNMT_E126_F	3.34E-01	D9	UGT1A1_P315_R	-7.13E-01		CALML3	4.38E-04		NLGN4X	1.15E-06		hsa-miR-19b	1.56E-04
	ESR2_P162_F	2.79E-01		ACVR1_P983_F	-6.84E-01		C5orf5	-5.20E-05	MR9	LAIR1	-9.08E-01		hsa-miR-19a	1.88E-05
	TRIP6_E33_F	1.75E-01		GFAP_P56_R	-1.42E-01		OR7C1	4.52E-05		MNDA	-4.18E-01	MI10	hsa-miR-518f	1.00E+00
	CTNNA1_P382_R	1.48E-01		KCNK4_E3_F	-5.01E-02		TRAK2	-1.80E-05		SLAMF8	-7.13E-04		hsa-miR-517b	8.67E-03
	FRZB_P406_F	9.94E-02		BLK_P14_F	-3.08E-02					FES	-1.98E-04			
	FES_P223_R	8.15E-02		SMARCB1_P220_R	2.36E-03					FCGR2B	-1.24E-05			
	GNMT_P197_F	7.90E-02		FGF7_P44_F	-5.73E-05					C1QB	-4.67E-06			
	IL17RB_E164_R	7.74E-02	D11	DNASE1L1_P39_R	-8.11E-01				MR10	HMGB3	-7.22E-01			
	ZMYND10_E77_R	5.49E-02		DKC1_P276_F	-4.77E-01					ECT2	-5.37E-01			
	PLAUR_E123_F	3.58E-02		DLG3_P62_R	-3.36E-01					C10orf18	-3.34E-01			
	FRZB_E186_R	2.18E-02		GLA_P112_F	-3.28E-02					DCLRE1A	-2.80E-01			
	TIMP3_seq_7_S38_F	3.47E-03		BIRC4_P122_R	-2.29E-03					PAPD1	-8.67E-04			
	PODXL_P1341_R	3.35E-03								BCL7A	-5.33E-04			
	LYN_P241_F	6.03E-04								TAF5	-1.35E-05			
	GLI3_P453_R	4.53E-04												
	PTCH2_P37_F	3.56E-04												
	MET_E333_F	1.78E-04												

Supplementary Table S2. List of variables in the first SPC

Variables are listed by the order of the absolute value of their factor loadings. Variables with high absolute factor loadings come first, and those with factor loadings close to 0, are listed at the end.

Cluster	Variables
**DNA**	
D1	S100A4_P887_R, DMP1_E194_F, TDGF1_E53_R, PLG_P370_F, GABRG3_E123_R, APOC1_P406_R, TDG_E129_F, SPI1_P48_F, XPC_P226_R, CTAG2_P1426_F, IL18BP_E285_F, NDN_E131_R
D2	NOS2A_P288_R, SPI1_P929_F, MMP7_P613_F, ITK_P114_F, IFNG_P188_F, TRPM5_E87_F, WRN_P969_F, ACTG2_E98_R, HLA-DOB_E432_R, GPR116_P850_F,HLA-DOB_P1114_R, EPHX1_P22_F, PMP22_P975_F, KLK11_P1290_F, KIAA0125_E29_F, IL4_P262_R, NID1_P677_F
D3	MUC1_E18_R, APBA2_P305_R, UGT1A7_P751_R, CPA4_P1265_R
D4	ALK_E183_R, KRAS_P651_F, IFNGR2_E164_F, RASA1_E107_F, CCKBR_P361_R, FGF9_P862_R, PTCH_E42_F, APC_E117_R, WNT5A_E43_F, EFNB3_E17_R, CDKN1C_P6_R, GAS1_P754_R, CDKN2B_E220_F
D5	FES_E34_R, FAS_P65_F, GNMT_E126_F, ESR2_P162_F, TRIP6_E33_F, CTNNA1_P382_R, FRZB_P406_F, FES_P223_R, GNMT_P197_F, IL17RB_E164_R, ZMYND10_E77_R, PLAUR_E123_F, FRZB_E186_R, TIMP3_seq_7_S38_F, PODXL_P1341_R, LYN_P241_F, GLI3_P453_R, PTCH2_P37_F, MET_E333_F
D6	BCL3_E71_F, COL6A1_P425_F, MCAM_P265_R, INHA_P1144_R, CD34_E20_R,EPS8_E231_F, IGSF4C_E65_F, BCAM_P205_F, KLF5_E190_R, LAMB1_E144_R, ITGB1_P451_F, PCGF4_P760_R, FGFR1_E317_F, DAB2IP_E18_R, KLF5_P13_F
D7	GATA6_P21_R, BMP3_E147_F, PENK_P447_R, FLT3_E326_R, ISL1_P379_F, NPY_P91_F, NPY_E31_R, NEFL_P209_R, TUSC3_E29_R, FZD9_P175_F, DSP_P36_F, ZNF264_P397_F, TPEF_seq_44_S88_R, RASGRF1_E16_F
D8	CD2_P68_F, BMP4_P123_R, TNFSF8_P184_F, CEACAM1_E57_R, PECAM1_P135_F, DLC1_E276_F, SEPT9_P374_F, SLC22A18_P216_R, LCN2_P141_R, HHIP_P578_R, PTHLH_P15_R
D9	UGT1A1_P315_R, ACVR1_P983_F, GFAP_P56_R, KCNK4_E3_F, BLK_P14_F, SMARCB1_P220_R, FGF7_P44_F
D10	PECAM1_E32_R, ERN1_P809_R, GP1BB_P278_R, CSF1R_E26_F, AFF3_P122_F, CASP8_E474_F, TNFSF8_E258_R, MAP3K8_P1036_F, HPN_P374_R, SIN3B_P607_F
D11	DNASE1L1_P39_R, DKC1_P276_F, DLG3_P62_R, GLA_P112_F, BIRC4_P122_R
D12	CPA4_P961_R, SRC_E100_R, THPO_P585_R, LMTK2_P1034_F
**mRNA**	
MR1	C6orf134, NCAM1, IL15RA, TRH, SFTPB, AGER, GPR4, SLIT3, ICAM2, CBR4, SULT1A2, IKBKE, GGA2, GCH1, GLS, SLC6A4
MR2	PSMD12, ZNF207, VDAC1, IMMT, HAT1, ZBTB11, WDR12, ALG8
MR3	CCT4, POLE3, DIMT1L, POLR3K, UTP11L, NUP37, COX7A2L, TFB2M, GTF2B, CKLF, MRPL9, EMG1
MR4	GLP1R, NCR2, KIAA0953, SLC9A3, CNGB1, AVPR1A, MYLC2PL, NMUR1, BTNL8, FUT6, PRO1768, GNRHR, ADRB3, PCTK2, PDCL3, CALML3, C5orf5, OR7C1, TRAK2
MR5	PTPRZ1, EPB41L4B, JAM3, GRIA3, MAPK13, RBM35B, SOX2, SPINT1, CLIP3, CLDN3, BHLHB9, ABHD4, BAI3, LLGL2, PSRC1, NLGN4X
MR6	KIDINS220, ZNF532, PABPN1, KHDRBS1, WIPF2, RNF126, PRPF40A, PEBP1,PUM1, SCRN1, SYNJ1, CHMP1A, BOP1
MR7	STT3A, PDIA6, AGPS, ITCH, KIAA0692, GANAB, NIPBL, DNAJC7, NRBP1, NCL, HYOU1
MR8	DUSP3, TPP1, LRP10, TRIP6, COPZ2, LOX, TXNDC15, ACTN1, IFITM3, FAM46A, SRPX2, RNASE4
MR9	LAIR1, MNDA, SLAMF8, FES, FCGR2B, C1QB

MR10	HMGB3, ECT2, C10orf18, DCLRE1A, PAPD1, BCL7A, TAF5
**miRNA**	
MI1	hsa-miR-553, hsa-miR-544, hsa-miR-384, ebv-miR-BART15, hsa-miR-626, hsa-miR-33b, hsa-miR-591, hsa-miR-802, hsa-miR-518d
MI2	hsa-miR-609, hsa-miR-595, ebv-miR-BART16, hsa-miR-647
MI3	hsa-miR-602, kshv-miR-K12-9, hsa-miR-563
MI4	hsa-miR-765, hsa-miR-188, hsa-miR-198, hsa-miR-630, hsa-miR-671, hsa-miR-100, hsa-miR-622, hsa-miR-583, hsa-miR-560, hsa-miR-638, hsa-miR-557, hsa-miR-572, hsv1-miR-H1, hsa-miR-575, hsa-miR-659
MI5	hsa-miR-648, ebv-miR-BART8-3p, hsa-miR-604, kshv-miR-K12-2, hsa-miR-658, hsa-miR-629, hsa-miR-639, ebv-miR-BART17-5p
MI6	hcmv-miR-US25-2-5p, hsa-miR-128a, hsa-miR-128b, hcmv-miR-UL36
MI7	hsa-miR-17-5p, hsa-miR-106a, hsa-miR-20a, hsa-miR-20b, hsa-miR-19b, hsa-miR-19a
MI8	hsa-miR-377, hsa-miR-376a
MI9	hsa-miR-429
MI10	hsa-miR-518f, hsa-miR-517b
